# Physical symptoms and brain morphology: a population neuroimaging study in 12,286 pre-adolescents

**DOI:** 10.1038/s41398-023-02528-w

**Published:** 2023-07-12

**Authors:** Fernando Estévez-López, Hannah H. Kim, Mónica López-Vicente, Jeroen S. Legerstee, Manon H. J. Hillegers, Henning Tiemeier, Ryan L. Muetzel

**Affiliations:** 1 Department of Social and Behavioral Sciences, Harvard T. H. Chan School of Public Health, Boston, MA USA; 2 Department of Child and Adolescent Psychiatry/Psychology, Erasmus MC University Medical Center, Rotterdam, The Netherlands; 3 Department of Education, Faculty of Education Sciences, SPORT Research Group (CTS-1024) and CERNEP Research Center, University of Almería, Almería, Spain; 4 Department of Radiology and Nuclear Medicine, Erasmus MC University Medical Center, Rotterdam, the Netherlands

**Keywords:** Molecular neuroscience, Psychiatric disorders

## Abstract

Physical symptoms, also known as somatic symptoms, are those for which medical examinations do not reveal a sufficient underlying root cause (e.g., pain and fatigue). The extant literature of the neurobiological underpinnings of physical symptoms is largely inconsistent and primarily comprises of (clinical) case-control studies with small sample sizes. In this cross-sectional study, we studied the association between dimensionally measured physical symptoms and brain morphology in pre-adolescents from two population-based cohorts; the Generation R Study (*n* = 2649, 10.1 ± 0.6 years old) and ABCD Study (*n* = 9637, 9.9 ± 0.6 years old). Physical symptoms were evaluated using continuous scores from the somatic complaints syndrome scale from the parent-reported Child Behavior Checklist (CBCL). High‐resolution structural magnetic resonance imaging (MRI) was collected using 3-Tesla MRI systems. Linear regression models were fitted for global brain metrics (cortical and subcortical grey matter and total white matter volume) and surface-based vertex-wise measures (surface area and cortical thickness). Results were meta-analysed. Symptoms of anxiety/depression were studied as a contrasting comorbidity. In the meta-analyses across cohorts, we found negative associations between physical symptoms and surface area in the (i) left hemisphere; in the lateral orbitofrontal cortex and pars triangularis and (ii) right hemisphere; in the pars triangularis, the pars orbitalis, insula, middle temporal gyrus and caudal anterior cingulate cortex. However, only a subset of regions (left lateral orbitofrontal cortex and right pars triangularis) were specifically associated with physical symptoms, while others were also related to symptoms of anxiety/depression. No significant associations were observed for cortical thickness. This study in preadolescents, the most representative and well-powered to date, showed that more physical symptoms are modestly related to less surface area of the prefrontal cortex mostly. While these effects are subtle, future prospective research is warranted to understand the longitudinal relationship of physical symptoms and brain changes over time. Particularly, to elucidate whether physical symptoms are a potential cause or consequence of distinct neurodevelopmental trajectories.

## Introduction

Physical symptoms, also known as somatic symptoms, are defined as symptoms for which a medical examination does not reveal a sufficient underlying root cause [1]. Physical symptoms are related to the presence of pain, fatigue and functional disturbances in organ systems such as dizziness or bowel symptoms [2]. These symptoms are prevalent in children and adolescents (hereinafter referred to as young people), with estimates as high as 20% [3]. Physical symptoms become persistent and disabling in 5% of cases, which imposes a burden on individuals, families and society. Importantly, persistent physical symptoms in young people are related to increased health care costs [4] and school absences [5]. Although it is widely speculated that brain plays a role in physical symptoms, little evidence is available in young people. Therefore, to identify neurobiological correlates of physical symptoms is of interest to help facilitate early diagnosis and the development of targeted interventions.

To detect possible neurobiological correlates of persistent physical symptoms researchers have repeatedly studied brain morphology using neuroimaging methods, such as magnetic resonance imaging (MRI) [6, 7]. To date, most of the previous structural brain imaging studies were conducted in relatively small clinical samples of adults. In general, results did not replicate in subsequent research in independent samples [8], although there are a number of exceptions. In comparison to peers without symptoms, young people with persistent physical symptoms have shown less grey matter volume in the prefrontal cortex [9], cingulate cortex [8, 9] and motor cortex [10]. The prefrontal and cingulate cortices are related to cognitive, emotional and behavioural regulation, which is key in the adaptation to physical symptoms [11, 12]. The motor cortex is involved in the control of voluntary movement [13]. Importantly, the function of these anatomical regions correspond well with difficulties that young people frequently experience in living with physical symptoms [14–16].

While previous findings shed light on the elusive link between physical symptoms and the brain, available research has relied on relatively small samples of adults with and without a clinical diagnosis of a specific physical symptom disorder (i.e., case-control studies). However, the severity of physical symptoms lies on a continuum in the general population [2, 17–19]. Thus, a dimensional approach may be useful in exploring neurobiological features associated with these symptoms in the general population. Also, it is well-known that the level of comorbidity between physical symptoms and symptoms of anxiety/depression is high [8, 20, 21]. However, most of the previous literature examining the associations between physical symptoms and brain structure failed to account for symptoms of anxiety/depression, which does not allow for disentangling the specificity of the past findings. In this context, large population-based cohort studies may help to overcome limitations in the previous literature and to identify generalisable associations between physical symptoms and brain features.

Therefore, the aim of this cross-sectional population-neuroimaging study was to examine the association between dimensional physical symptoms and brain structure in pre-adolescents from the general population. Importantly, we investigated this aim in two large, independent population-based cohorts to improve the generalisability of our results. Most of the previous literature on brain morphology focused on brain volume, which is the product of cortical thickness and surface area. Thus, here we examined surface area and thickness separately in order to disentangle whether one or both components were implicated. It should be noted that cortical thickness and surface are two ontogenetically and genetically distinct characteristics of the cortex [22] resulting from different growth processes and trajectories [23]. For instance, cortical thickness has been shown to be impacted by underlying processes such as arborisation/pruning within grey matter, while surface area is driven by the division of progenitor cells in the embryological periventricular area [24]. In the present study, we conducted surface-based vertex-wise analyses because they provide a more accurate measurement of brain morphology in comparison to traditional methods [25, 26]. We hypothesised associations with specific regions but not with global brain structure metrics [8–10]. Particularly, our hypothesis are that more physical symptoms are associated with a thinner cortex or a smaller surface area in the following candidate regions highlighted in the extant literature: the prefrontal cortex [9], cingulate cortex [8, 9] and the motor cortex [10].

## Methods

### Design

This cross-sectional study used data from two independent population-based cohorts: (i) the Generation R Study, the Netherlands and (ii) Adolescent Brain Cognitive Development (ABCD) Study, the United States of America (USA).

### Participants

**Fig. 1:**
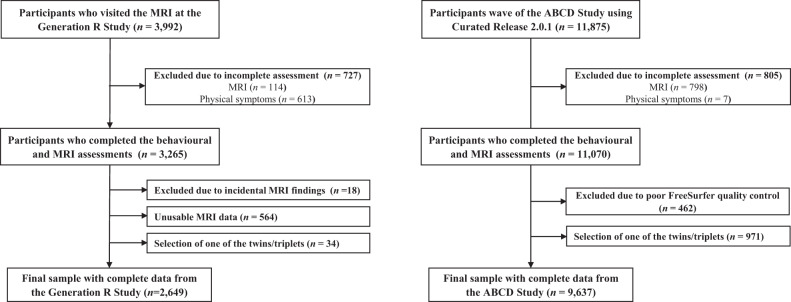
Flowchart of participants. ABCD Adolescent Brain Cognitive Development. MRI magnetic resonance imaging.

The Generation R Study is a population-based cohort of maternal and child health from foetal life onward based in Rotterdam (the Netherlands). A detailed description of the Generation R Study is available elsewhere [27]. Briefly, between March 2013 and November 2015, participants aged 8–12 years visited a study‐dedicated research centre for a detailed behavioural assessment and also underwent MRI scan [28]. Of the 3992 children who visited our research centre, 3265 participants had data on both physical symptoms and MRI. After excluding those participants with incidental findings (*n* = 18) and unusable MRI data (*n* = 564) as well as randomly selecting one participant from twins and triplets (34 participants were excluded), the final sample for statistical analyses consisted of 2649 participants. Figure 1 illustrates the exclusions in detail. The Medical Ethics Committee of the Erasmus Medical Center approved all study procedures, and all participants provided written informed consent or assent.

The ABCD Study is a population-based cohort study of brain development and child health across 21 sites in USA. We used data from the baseline assessment (release 2.0.1) of the ABCD Study [29]. An extensive description of the study is provided elsewhere [29, 30]. The baseline cohort of the ABCD study includes data from 11,875 children between 9 and 10 years of age. Of them, 11,070 participants have data on both physical symptoms and MRI. Four hundred and sixty-two participants were excluded because of MRI poor quality data. Among twins and triplets, one participant from each family was randomly selected to be included in the study population (971 participants were excluded). Thus, the final sample consisted of 9637 participants. Figure 1 illustrates the flowchart of participants. Centralised institutional review board (IRB) approval was obtained from the University of California, San Diego, USA. Study sites obtained approval from their local IRBs. Written, informed consent and assent were provided by each parent and child, respectively.

### Physical symptoms

In both the Generation R and ABCD studies, the parent-reported somatic complaints syndrome subscale from the school-age version (for ages 6–18) of the Child Behavior Checklist (CBCL) was used to assess child physical symptoms [31]. The CBCL is a validated and reliable inventory that uses caregiver-reported information to assess behavioural problems in children [32]. The Dutch version of the CBCL was used in the Generation R study, this version is valid and reliable (e.g., Cronbach’s α for somatic complaints = 0.74) [33, 34]. The caregivers rated behaviour problems of the child in the previous 6 months using a three-point Likert scale (0 = not true, 1 = somewhat true, 2 = very true). The CBCL is composed of 7 subscales. The somatic complaints subscale includes 11 items assessing the presence of the following signs: (i) nightmares, (ii) constipation, doesn’t move bowels, (iii) dizziness, (iv) overtiredness without a good reason and (v) physical problems without known medical cause: headaches, stomach aches, other aches/pain, nausea/feels sick, problems with eyes (not if corrected by glasses), rashes/skin problems and vomiting/throwing up. The scores of the CBCL somatic complaints subscale range from 0 to 22, with higher scores indicating more physical symptoms.

### Neuroimaging: brain structure

Detailed descriptions of the scan protocol, imaging procedures and subsequent processing of the imaging data of the Generation R [28, 35] and ABCD [36, 37] studies are available. Briefly, both studies performed high‐resolution structural MRI that were acquired using 3-Tesla MRI systems. Data quality assurance consisted of a multi-step process including both manual review by trained technicians and automated software [35, 37]. Data from both cohorts were processed through FreeSurfer (version 6.0) on the same high-performance computing system. Surface-based maps of surface area and cortical thickness were co-registered to a common stereotactic space, and smoothed with a 10 mm full width half max Gaussian kernel. Further details are provided in supplementary materials (Section A).

### Potential confounders

Potential confounders of the present study were included according to availability in cohorts and identified according to previous literature (referenced below after each variable). We considered the following variables based on previous research showing associations with both physical symptoms and brain morphology: age, sex, national origin for Generation R and race/ethnicity for ABCD, maternal education for Generation R and parental education for ABCD, monthly household income [38], body mass index (BMI) [39, 40] and non-verbal intelligence quotient (IQ) [41, 42]. Maternal/parental education and household income were considered proxies of socioeconomic status. In ABCD, we additionally adjusted models for the 21 study sites.

Further details regarding the assessment of potential confounders are provided in supplementary materials (Section A).

### Statistical analyses

Analyses were run using the R statistical software (version 3.4.3). For the global brain structure metrics (i.e., cortical and subcortical grey matter and total white matter volume), we conducted a set of separate linear regression models with brain structure as the independent variable and physical symptoms (continuous somatic complaints syndrome subscale from the CBCL, square root transformed) as the dependent variable. The models were adjusted for intracranial volume, age at MRI, sex, parental national origin or race/ethnicity, maternal/paternal education, household income, BMI and IQ.

Surface-based vertex-wise analyses were conducted using the R package QDECR [43], which was developed to support population neuroimaging research (e.g., accounting for diverse (imputed) confounders and analysing large samples). Further information about the advantages of the R package QDECR is available elsewhere [43]. At each cortical vertex, we examined the association of cortical thickness and surface area with physical symptoms. Models for cortical thickness and surface area included the same covariates as the model for the global brain structure described above, except that intracranial volume was not included. Resulting *p*-value maps were corrected for multiple comparisons at the vertex level using Gaussian Monte Carlo Simulations [44]. We set the cluster forming threshold to 0.001, as this has shown high correspondence with actual permutation testing across all surface measures [45]. We further applied Bonferroni correction to account for analysing both hemispheres separately (i.e., *p* < 0.025 cluster-wise). Surface-based vertex-wise analyses were conducted first in each cohort separately. Finally, the results from both cohorts were pooled using fixed-effects meta-analysis (R package ‘meta’). *P*-values from meta analyses were corrected for multiple comparisons using False Discovery Rate (FDR). Supplemental analyses, using symptoms of anxiety/depression as a contrasting comorbidity, were conducted when significant findings were found for the association between physical symptoms and brain structure in order to determine whether the findings were specific for physical symptoms or overlapping with those from symptoms of anxiety/depression. Our rationale was that the level of comorbidity between physical symptoms and symptoms of anxiety/depression is high but the role (e.g., confounder, mediator or collider) of the latter in the association between brain structure and physical symptoms is unclear [8, 20, 21]. Importantly, we have previously showed that adjusting for variables that may not be a confounder may result in introducing bias in the results [46]. Thus, as symptoms of anxiety/depression are unlikely simple confounders, we explored these symptoms in the context of physical symptoms. Details regarding the assessment of symptoms of anxiety/depression are provided in supplementary materials (Section A).

In additional analyses, we re-ran the primary analyses (i.e., continuous scores of physical symptoms) in the ABCD study using a less conservative multiple testing correction threshold (cluster forming threshold = 0.005 rather than 0.001) because the ABCD study comprises a substantial proportion of the sample in the meta-analysis. In sensitivity analyses, we re-ran all the models with dichotomous scores of physical symptoms, which is similar to the previous case-control literature involving clinical diagnosis. The dichotomisation was based on the borderline clinical cut-off score of physical symptoms; i.e., >93^rd^ percentile [31]. Due to the potential loss of statistical power of this dichotomisation, the associations were tested using two thresholds of significance: (i) accounting for multiple comparisons, as in the main analyses and (ii) a more liberal alternative (*p*_uncorrected_ < 0.001).

Missing covariate data were observed: (i) in the Generation R Study for parental national origin, BMI, maternal education (all, ≤1%) and monthly household income (12%) and non-verbal IQ (12%) and (ii) in the ABCD study for BMI, race/ethnicity, parental education (all, ≤1%) and non-verbal IQ (2%). Missing covariate data were estimated by multiple imputation with the R package MICE [47]. With 100 iterations, a total of 40 imputed datasets were generated, and results were pooled using Rubin’s rules [48].

## Results

The total sample of the present study comprised 12,286 participants. Table 1 shows the characteristics of 2649 participants from the Generation R study (10.1 ± 0.6 years old). Table 2 shows the characteristics of 9637 participants from the ABCD study (9.9 ± 0.6 years old). In both cohorts, half of the participants (*n* = 5921) were female (1335 (50%) in the Generation R Study and 4586 (48%) in the ABCD study) and most frequently from families with high income (1350 (51%) in the Generation R Study and 3632 (38%) in the ABCD study).

**Table 1:** Characteristics of the participants in the Generation R study (*n* = 2,649).

	Mean	SD
Age	10.1	0.6
Body mass index	17.4	2.5
Non-verbal intelligence quotient	103.9	14.7
Physical symptoms (CBCL)	1.5	2.0
	***n***	**%**
Sex		
Female	1335	50.4
National origin		
Dutch	1721	65.0
Caribbean	223	8.4
Non-Dutch Western	223	8.4
Moroccan or Turkish	212	8.0
African	123	4.6
American or Asian	108	4.1
Indonesian	15	0.6
Missing data	24	0.9
Maternal education level		
No/Primary/Secondary studies	1746	65.9
Higher education	873	33.0
Missing data	30	1.1
Monthly household income		
≤ €2,000	380	14.3
> €2,000 to ≤ €3,200	604	22.8
> €3,200	1350	51.0
Missing data	315	11.9

Non-imputed data are shown.

*CBCL* the Child Behavior Checklist, *SD* Standard Deviation.

**Table 2:** Characteristics of the participants in the Adolescent Brain Cognitive Development (ABCD) study (*n* = 9637).

	Mean	SD
Age	9.9	0.6
Body mass index	18.8	4.0
WISC-V Matrix Reasoning Total Score	9.9	3.0
Physical symptoms (CBCL)	1.5	2.0
	***n***	**%**
Sex		
Female	4586	47.6
Race/ethnicity		
White	4976	51.6
Black	1416	14.7
Hispanic	2047	21.2
Asian	210	2.2
Other	976	10.1
Missing data	12	0.1
Highest parental education		
<High School Diploma	482	5.0
High School Diploma/GED	938	9.7
Some College	2517	26.1
Bachelor	2400	24.9
Post Graduate Degree	3289	34.1
Missing data	11	0.1
Annual household income		
Low (<$50,000)	2669	27.7
Middle (≥$50,000 to < $100,000)	2509	26.0
High (≥$100,000)	3632	37.7
Missing data	827	8.6

Non-imputed data are shown.

*CBCL* the Child Behavior Checklist, *SD* Standard Deviation, *GED* General Educational Development, *WISC-V* the Wechsler Intelligence Scale for Children - Fifth Edition.

Table 3 shows the association between physical symptoms and the global metrics of brain structure. In general, the unstandardised regression coefficients were small and all were non-significant. For example, in the Generation R Study, for every unit increase in (square root transformed) physical symptoms, there was a 6.3 × 10^−7^ mm^3^ decrease in cortical grey matter volume (standard error (SE) = 6.5 × 10^−7^, *p*_uncorrected_ = 0.3, *p*_FDR_ = 0.8). Similarly, for ABCD, for every unit increase in physical symptoms (square root transformed), there was a 4.1 × 10^−7^ mm^3^ decrease in total white matter volume (SE = 3.2 × 10^−7^, *p*_uncorrected_ = 0.2, *p*_FDR_ = 0.8). Supplementary figure S1 shows that there were no statistically significant associations between the global metrics of brain structure and physical symptoms in the meta-analysis of the two studies (all, *p*-values ≥ 0.4).

**Table 3:** Associations of brain structure with physical symptoms.

Generation R study (*n* = 2649)	Cortical grey matter volume (mm^3^)				Subcortical grey matter volume (mm^3^)				Total white matter volume (mm^3^)			
	*b*	SE	*p*_unc_	*p*_FDR_	*b*	SE	*p*_unc_	*p*_FDR_	*b*	SE	*p*_unc_	*p*_FDR_
Physical symptoms	−6.3 × 10^−7^	6.5 × 10^−7^	0.337	0.755	−2.2 × 10^−6^	6.0 × 10^−6^	0.716	0.755	−4.5 × 10^−7^	7.5 × 10^−7^	0.551	0.755

Adolescent Brain Cognitive Development (ABCD) study (*n* = 9637)	Cortical grey matter volume (mm^3^)				Subcortical grey matter volume (mm^3^)				Total white matter volume (mm^3^)			
	*b*	SE	*p*_unc_	*p*_FDR_	*b*	SE	*p*_unc_	*p*_FDR_	*b*	SE	*p*_unc_	*p*_FDR_
Physical symptoms	−8.1 × 10^−8^	2.6 × 10^−7^	0.755	0.755	−2.1 × 10^−6^	3.2 × 10^−6^	0.510	0.755	-4.1 x10-^7^	3.2 × 10−^7^	0.199	0.755

*b* Unstandardised Coefficient Regression, *SE* Standard Error, *unc* uncorrected, *FDR* False Discovery Rate.

The model was adjusted for age, sex, national origin (Generation R) or race/ethnicity (ABCD), estimated intracranial volume, maternal education (Generation R) or parental education (ABCD), household income, body mass index and non-verbal intelligence quotient. ABCD analyses were additionally adjusted for the 21 study sites. Physical symptoms were assessed using the school-age version (for ages 6–18) of the Child Behavior Checklist (CBCL).

In order to determine whether there were any focal associations between physical symptoms and brain structure, we used a step-wise approach. First, whole-brain vertex wise analyses were conducted in each cohort separately. In the individual cohorts, we did not observe any associations between physical symptoms and brain structure (i.e. surface area and cortical thickness), after adjusting for multiple comparisons. Supplementary table 1 shows the range of unstandardized regression coefficients for each individual cohort, in order to depict the overall magnitude of association. Second, we pooled the vertex-wise results (regression coefficients and standard errors) from both cohorts using a meta-analysis. We found significant associations (*p*_FDR_ < 0.05*)* between physical symptoms and surface area but not cortical thickness in the vertex-wise meta-analysis. The summary data and code to generate figures showing the association between continuous scores of physical symptoms and surface area can be found in https://github.com/FerEstevezLopez/doi_10.1038-s41398-023-02528-w.

**Fig. 2:**
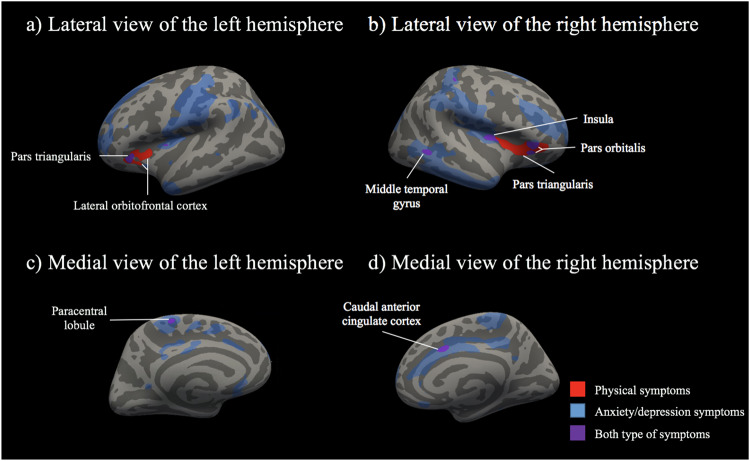
Significant associations between surface area and continuous scores of physical symptoms, anxiety/depression symptoms and both types of symptoms from meta-analyses after correction for multiple testing (*n* = 12,286). Significant clusters of <45 mm^2^ are not annotated in the figure because they were considered exceedingly small. Specific findings for anxiety/depression symptoms are not annotated because are not the primary focus of this work. The models were adjusted for age, sex, national origin (Generation R) or race/ethnicity (ABCD), estimated intracranial volume, maternal education (Generation R) or parental education (ABCD), household income, body mass index and non-verbal intelligence quotient. ABCD analyses were additionally adjusted for the 21 study sites. Physical symptoms were assessed using the school-age version (for ages 6–18) of the Child Behavior Checklist (CBCL). The cluster annotated as “lateral orbitofrontal cortex” in the left hemisphere also included regions in the insula, pars triangularis and pars orbitalis. The cluster annotated as “pars triangularis” in the right hemisphere also included regions in the insula, lateral orbitofrontal cortex and pars orbitalis. For interpretation of the references to colour in this figure legend, the reader is referred to the web version of this article.

Figure 2 shows that some of the significant associations in surface area were specific for physical symptoms (i.e., they did not overlap with those found for symptoms of anxiety/depression). These associations were found in the prefrontal cortex; in particular, a cluster of 640 mm^2^ in the right hemisphere including the pars triangularis, insula, lateral orbitofrontal cortex and pars orbitalis, and two spatially-close clusters of 229 mm^2^ and 107 mm^2^ in the left hemisphere including the lateral orbitofrontal cortex, insula, pars triangularis and pars orbitalis. We also found overlapping associations of physical symptoms and symptoms of anxiety/depression with surface area. In the right hemisphere, these overlapping associations were mostly in the pars orbitalis (a cluster of 156 mm^2^), insula (a cluster of 105 mm^2^), middle temporal gyrus (a cluster of 84 mm^2^) and caudal anterior cingulate cortex (a cluster of 84 mm^2^). In the left hemisphere, these overlapping associations were mostly in the pars triangularis (a cluster of 126 mm^2^).

### Additional and sensitivity analyses

In additional analyses, we observed that the largest clusters replicated when primary analyses were re-run only in the ABCD study using a more liberal threshold (Supplementary fig. S2). In sensitivity analyses, the findings observed in the primary analyses remained largely unchanged when using dichotomised scores of physical symptoms and a liberal threshold of significance (*p*_uncorrected_ < 0.001; Supplementary fig. S3). However, consistent with a loss of statistical power, this result did not remain after correction for the stringent multiple comparisons threshold applied to the main analyses.

## Discussion

In this population neuroimaging study, we leveraged a dimensional approach to physical symptoms in two independent and large cohorts of pre-adolescents. We aimed to examine the association between physical symptoms and brain structure. In the meta-analysis of both cohorts, we found subtle associations between more physical symptoms and less surface area in the (i) left hemisphere; in the lateral orbitofrontal cortex and pars triangularis and (ii) the right hemisphere; in the pars triangularis, the pars orbitalis, insula, middle temporal gyrus and caudal anterior cingulate cortex. However, only few regions located in the prefrontal cortex (left lateral orbitofrontal cortex and right pars triangularis) were specifically associated with physical symptoms, while others were also related to symptoms of anxiety/depression. Sensitivity analyses, using dichotomised scores of physical symptoms, complement the continuous analyses and offer additional evidence for the dimensional nature of physical symptoms in the general population.

In the present study, most of the specific associations between dimensional physical symptoms and surface area were in regions of the prefrontal cortex; namely, in the lateral orbitofrontal cortex and pars triangularis (left hemisphere) as well as in the pars triangularis and pars orbitalis (right hemisphere). Briefly, these regions are involved in cognitive, emotional and behaviour regulation, key processes in adaptation to physical symptoms [11, 12, 49, 50]. Additionally, previous literature has robustly identified that, in comparison to healthy controls, adults [8] and young people [9] with physical symptoms disorders have lower grey matter volume in the prefrontal cortex. Importantly, the previous literature did not account for symptoms of anxiety/depression. For the first time, our study showed that the associations between more dimensional physical symptoms and lower surface area were specific for the lateral orbitofrontal cortex (left hemisphere) and pars triangularis (right hemisphere).

While the cross-sectional design of previous investigations, including the present study, precludes drawing conclusions about the directionality of the specific association between physical symptoms and brain morphology, we speculate that experiencing physical symptoms may result in subtle modifications in surface area. In this line, the orbitofrontal cortex is involved in inhibition of chronic pain [51]. Also, the repeated experience of physical symptoms may lead to lower surface area in modulatory areas of physical symptoms including the orbitofrontal cortex [52]. Alternatively, surface area features could precede physical symptoms. A lower surface area of the pars triangularis, a brain region involved in language processing, may be linked to difficulties in verbally expressing emotions which has the potential to induce distress, manifesting as physical symptoms [53–55]. Overall, further longitudinal research is needed to test the directionality of our findings.

The present study showed overlapping associations between more dimensional physical symptoms or more symptoms of anxiety/depression with smaller surface area in regions of the right hemisphere; particularly, in the caudal anterior cingulate cortex, insula and middle temporal gyrus. Of them, we had hypothesis only about the anterior cingulate cortex because previous literature robustly found that, in comparison to healthy controls, adults [8] and young people [9] with physical symptoms disorders have lower grey matter volume in this region. The anterior cingulate cortex is involved in emotional and cognitive aspects of physical symptoms such as the level of unpleasantness of the experienced symptoms and the formation of fear memories [56]. Moreover, the present study identified overlapping associations of more physical symptoms or more symptoms of anxiety/depression and less surface-area of non-hypothesised regions in the right hemisphere; in particular, in the insula and middle temporal gyrus. The functions of these regions include semantic memory as well as integration of sensory and motor information (among others, pain), which are often impaired in people with physical symptoms [11, 57–59]. These regions previously emerged in the literature but findings did not replicate in subsequent research [8]. Thus, using data from two independent and large cohorts, the present well-powered study provided robust evidence of associations between physical symptoms and the surface area of the insula and middle temporal gyrus, indicating that these associations are also observed for symptoms of anxiety/depression.

Although in the present study we found an association between more dimensional physical symptoms and smaller surface area of the motor cortex (left hemisphere), the size of the cluster in this region was considerably small (i.e., only 9 mm^2^). Additionally, more symptoms of anxiety/depression were associated with smaller surface area in a relatively large cluster of the motor cortex in the left hemisphere (i.e., 4077 mm^2^), which is in line with previous literature [60]. Thus, findings of the present study may help to overcome inconsistencies in the previous literature [9, 10]. In particular, our results are in line with previous studies concluding no association between physical symptoms and the structure of the motor cortex [9] seeming plausible that associations previously observed between physical symptoms and this region [10] may be driven by symptoms of anxiety/depression [61].

A number of features of the present study allowed us to contribute to the current literature. First, it is widely accepted that physical symptoms lie on a continuum in the general population [2, 17–19]. However, scarce investigation has focused on the association between dimensionally assessed physical symptoms and brain structure in the general population. Indeed, previous studies followed a case-control design focusing on paediatric clinical samples such as irritable bowel syndrome [9] or persistent gastrointestinal symptoms [62]. In the present study, participants were recruited from the general population, which likely results in more generalisable findings. Additionally, our dimensional conceptualisation to physical symptoms, accounting for the full spectrum of these symptoms, allowed us to better understand the associations under study. Importantly, sensitivity analyses using dichotomous scores of physical symptoms yielded to null findings using strict multiple testing correction. We speculated that these null findings were because splitting participants in two groups negatively impacted our statistical power. Accordingly, we used a more liberal threshold of significance (*p*_uncorrected_ < 0.001), which resulted in the replication of the findings from our primary dimensional analyses. Thus, the present study provided evidence supporting dimensional conceptualisations of physical symptoms, which is in line with the research domain criteria initiative by the National Institute of Mental Health (see, https://www.nimh.nih.gov/research/research-funded-by-nimh/rdoc).

Second, previous literature used small samples. In this study, we included two large and independent cohorts. Each of the cohorts involved more than 2000 participants, which is in line with the most recent rule-of-thumb recommendations to ensure the robustness and replicability of associations between behaviour and brain [63, 64]. In line with the underpowered correlation paradox [63], the magnitude of the significant associations found in the present study was subtle and smaller than previously reported from small sample size studies. To have confirmed that there is an association between physical symptoms and subtle alterations of surface area in pre-adolescents from the general population is of relevance for, at least, two particular reasons [64]. First, physical symptoms are better understood as a complex trait in which a combination of many small effect contributions are expected rather than large contributions for a few factors [65]. Second, these subtle effects may accumulate over childhood and adolescence imposing a burden for future health such as the development of disorders characterised by the presence of physical symptoms in adulthood [66].

Several limitations merit discussion in the present study. First, our cross-sectional design did not allow us to test the temporality of the associations under study. It is of interest to determine whether (i) experiencing more physical symptoms impacts in the structure of the brain or (ii) brain structure differences lead to experiencing physical symptoms. Thus, further prospective research is warranted, particularly with several repeated assessments and with sufficient follow-up time. Second, the narrow range of age precludes the generalisation of the findings to younger or older ages. Third, similar to most of the epidemiological studies [67], we evaluated the presence of the symptoms but not their impact on daily life including, for example, school absence, academic performance, and social and physical activities. A more comprehensive assessment of physical symptoms may help to better understand the associations under study. Fourth, we used parent-reported data on physical symptoms which, in comparison to structure interviews, may introduce measurement error or bias [68]. Several strengths of this study also deserve mention, most notably the inclusion of two, large population-based cohorts. Additional strengths were, first, the cohorts were well harmonised. Particularly, the same questionnaire was used to assess physical symptoms along a continuum and similar methods were used to measure and analyse brain structure. Second, adjustment for several important confounding factors was possible, avoiding overestimation of the associations between physical symptoms and brain morphology [69]. Third, this study involved typically developing children, which may help address selection bias inherent to clinical studies [69]. Fourth, cohorts are both ethnically diverse [70]. Thus, as opposed to the previous literature in the field of physical symptoms, the present study ensured a better generalisability of our findings in a broader context. Fifth, we determined the specificity of the associations between physical symptoms and brain structure, studying symptoms of anxiety/depression as a contrasting comorbidity.

## Conclusions

Pooling data from two independent and large cohorts including a total of 12,286 preadolescents from the general population and using a dimensional approach for measuring (early signs of) physical symptoms, we found subtle cross-sectional associations between more dimensionally assessed physical symptoms and less surface area of both the right and left hemispheres. Particularly, we found subtle associations between more physical symptoms and less surface area in the (i) left hemisphere; in the lateral orbitofrontal cortex and pars triangularis and (ii) the right hemisphere; in the pars triangularis, the pars orbitalis, insula, middle temporal gyrus and caudal anterior cingulate cortex. However, only few regions located in the prefrontal cortex (left lateral orbitofrontal cortex and right pars triangularis) were specifically associated with physical symptoms, whereas others were also related to symptoms of anxiety/depression. The robustness of these associations was confirmed in sensitivity analyses using dichotomised scores of physical symptoms. The functions of these anatomical regions correspond well with difficulties that young people frequently experience in living with physical symptoms (e.g., cognitive, emotional and behavioural regulation). This is the first population neuroimaging study of physical symptoms, that is representative of pre-adolescent populations in high income countries and is also well-powered. Future prospective research is warranted to understand the longitudinal relationship of the associations under study. In particular, to elucidate whether physical symptoms are potential causes or consequences of subtle brain modifications.

## Supplementary information

Supplementary materials

## Data Availability

The Generation R datasets generated and/or analysed during the current study are not publicly available due to legal and ethical regulations, but may be made available upon request to the Director of the Generation R Study, Vincent Jaddoe (v.jaddoe@erasmusmc.nl), in accordance with the local, national, and European Union regulations. The ABCD data repository grows and changes over time. The ABCD data used in this report came from the ABCD Data Release 2.0 (DOI: 10.15154/1503209, March 2019) and ABCD Fix Release 2.0.1 (DOI: 10.15154/1504041, July 2019). The summary data to generate figures showing the primary analyses on the association between continuous scores of physical symptoms and surface area can be found in: https://github.com/FerEstevezLopez/doi_10.1038-s41398-023-02528-w.

## Code availability

The Generation R datasets generated and/or analysed during the current study are not publicly available due to legal and ethical regulations, but may be made available upon request to the Director of the Generation R Study, Vincent Jaddoe (v.jaddoe@erasmusmc.nl), in accordance with the local, national, and European Union regulations. The ABCD data repository grows and changes over time. The ABCD data used in this report came from the ABCD Data Release 2.0 (DOI: 10.15154/1503209, March 2019) and ABCD Fix Release 2.0.1 (DOI: 10.15154/1504041, July 2019). The summary code to generate figures showing the primary analyses on the association between continuous scores of physical symptoms and surface area can be found in: https://github.com/FerEstevezLopez/doi_10.1038-s41398-023-02528-w.
