# Supplementary material for: Physical symptoms and brain morphology: a population neuroimaging study in 12,286 pre-adolescents
Source: Transl Psychiatry. 2023 Jul 12;13:254. doi: 10.1038/s41398-023-02528-w (PMC10338487; doi:10.1038/s41398-023-02528-w)
Supplement: Supplementary file 1 — Supplementary materials [file 41398_2023_2528_MOESM1_ESM.docx]

**Supplementary materials**

**Section A. Expanded methods**

**Image acquisition, processing and quality assurance**

*The Generation R Study*

Before scanning, children underwent a mock scanning session in order to familiarize them with the procedure and scanning environment. Then, the high‐resolution structural MRI was collected on a study-dedicated, 3-Tesla MRI system (MR-750W, General Electric, Milwaukee, WI, US) using an eight-channel, receive-only head coil. Data quality assurance was a multi-step process including both visual inspection and automated software. The high-resolution, T_1_-weigthed structural MRI data was acquired using a coronal inversion recovery fast spoiled gradient recalled sequence with the following parameters: GE option BRAVO, T_R_ = 8.77 ms, T_E_ = 3.4 ms, T_I_ = 600 ms, flip angle = 10°, matrix size = 220 × 220, field of view = 220 mm × 220 mm, slice thickness = 1 mm, number of slices = 230, ARC acceleration factor = 2. T1-weighted images were processed through the FreeSurfer analysis suite, version 6.0.0 [1].

*The ABCD Study*

The imaging protocol was developed by the ABCD Data Analysis and Informatics Center (DAIC) and the ABCD Imaging Acquisition Workgroup and was harmonized for all scanner platforms. Scanning occurred in one or two sessions and included 3D T1- and 3D T2-weighted images of brain structure. As previously detailed [2], imaging data collection occurred at 21 sites using a number of models of 3 tesla (3T) scanners from three different vendors: Siemens, General Electric, and Phillips. The specific scanner models used for data collection were General Electric Discovery MR750, Siemens Prisma, Siemens Prisma Fit, Phillips Achieva dStream, and Phillips Ingenia. More details on the imaging parameters are as follows: TR (repetition time) 2400 to 2500 ms; TE (echo time) 2 to 2.9 ms; FOV (field of view) 256 × 240 to 256; FOV phase of 93.75% to 100%; matrix 256 × 256; 176 to 225 slices; TI (inversion delay) 1060 ms; flip angle of 8°; voxel resolution of 1×1×1 mm; total acquisition time from 5 minutes 38 seconds to 7 minutes 12 seconds.

Thorough descriptions of the processing and analysis of brain data can be found elsewhere [3]. To summarize, DAIC performed centralized processing and analysis of the structural data using a collection of processing steps within the Multi-Modal Processing Stream (MMPS), which is a software package developed and maintained at the Center for Multimodal Imaging and Genetic (CMIG) at the University of California, San Diego (UCSD). Briefly, this pipeline included: 1) preprocessing (correction for gradient nonlinearity distortions, intensity scaling and inhomogeneity correction, registration to an averaged reference brain in standard space, and manual quality control (QC)); 2) brain segmentation (cortical surface reconstruction and subcortical segmentation performed based on automated, atlas-based, segmentation procedures in FreeSurfer v5.3); 3) derivation of morphometric measures (calculation of average volume in each cortical parcel of the standard FreeSurfer Desikan parcellation scheme [4] and in each subcortical region [5]); and finally, 4) post-processing QC (manual review by trained technicians for motion, intensity inhomogeneity, white matter underestimation, pial overestimation, and magnetic susceptibility artifact).

**Assessment of potential confounders**

*The Generation R Study*

Sex and age was obtained from midwife and hospital records at birth.

Paternal national origin of the child was based on the country of birth of both parents.

Monthly household income (≤€2,000, >€2,000 to ≤€3,200 and >€3,200) was reported by the primary caregiver.

Highest maternal educational level, reported by the primary caregiver, was categorised into high (higher education, phase 1 or 2) or low (no education finished, primary school, or secondary school).

Body Mass Index (BMI). Child height was measured in standing position using a Harpenden stadiometer (Holtain Limited, Crymych, United Kingdom), and weight was measured without heavy clothing and shoes using a mechanical personal scale (SECA). Height and weight were used to calculate the BMI (in kg/m^2^).

Non-verbal intelligence quotient (IQ). Participants completed two subtests of the Snijders-Oomen Non-Verbal Intelligence Test-Revised (SON-R 2½-7): “Mosaics,” a spatial visualization task, and “Categories,” an abstract reasoning task [6]. The raw scores were converted to IQ scores using age and sex-specific norms.

*The ABCD Study*

Demographic variables (including age and sex) were reported as part of the Parent Demographics Survey.

Child race/ethnicity was categorized as White, Black, Hispanic, Asian, Other.

Household combined income (<$50,000, >=$50,000 & < $100,000, >=%100,000) and highest parental education (high school diploma/GED, college, Bachelor degree, Postgraduate degree) were self-reported by the primary caregiver.

Body mass index was calculated based on an average of three standing height measurements and an average of three weight measurements.

The Wechsler Intelligence Scale for Children-6 Matrix Reasoning total scaled score was used to measure non-verbal IQ.

**Assessment of symptoms of anxiety/depression**

Parent-reported data on the anxious/depressed syndrome subscale from the school-age version (for ages 6 to 18) of the CBCL [7] was used in both cohorts.

**REFERENCES**

1. Fischl B. FreeSurfer. Neuroimage. 2012;62:774–781.

2. Casey BJ, Cannonier T, Conley MI, Cohen AO, Barch DM, Heitzeg MM, et al. The Adolescent Brain Cognitive Development (ABCD) study: Imaging acquisition across 21 sites. Dev Cogn Neurosci. 2018;32:43–54.

3. Hagler DJ, Hatton S, Cornejo MD, Makowski C, Fair DA, Dick AS, et al. Image processing and analysis methods for the Adolescent Brain Cognitive Development Study. Neuroimage. 2019;202:116091.

4. Desikan RS, Ségonne F, Fischl B, Quinn BT, Dickerson BC, Blacker D, et al. An automated labeling system for subdividing the human cerebral cortex on MRI scans into gyral based regions of interest. Neuroimage. 2006;31:968–980.

5. Fischl B, Salat DH, Busa E, Albert M, Dieterich M, Haselgrove C, et al. Whole brain segmentation: automated labeling of neuroanatomical structures in the human brain. Neuron. 2002;33:341–355.

6. Basten M, van der Ende J, Tiemeier H, Althoff RR, Rijlaarsdam J, Jaddoe VW V., et al. Nonverbal intelligence in young children with dysregulation: the Generation R Study. Eur Child Adolesc Psychiatry. 2014;23:1061–1070.

7. Achenbach T, Rescorla L. Manual for the ASEBA preschool forms and profiles. Burlington, VT: VT: University of Vermont, Research Center for Children, Youth, & Families; 2000.

**Section B. Supplementary table**

Supplementary table S1. The range of unstandardized regression coefficients for each surface-based vertex-wise measurement in each individual cohort.

|  |  | Generation R  (N=2,649) | | ABCD  (N=9,637) | |
| --- | --- | --- | --- | --- | --- |
|  |  | Min | Max | Min | Max |
| Right hemisphere | Surface area | -0.01127 | 0.00639 | -0.00674 | 0.00330 |
|  | Cortical thickness | -0.03648 | 0.02292 | -0.00747 | 0.10068 |
| Left hemisphere | Surface area | -0.01322 | 0.00666 | -0.00520 | 0.00429 |
|  | Cortical thickness | -0.02255 | 0.02174 | -0.00731 | 0.01047 |

*Note.* ABCD, Adolescent Brain Cognitive Development.

**Section C. Supplementary figures**


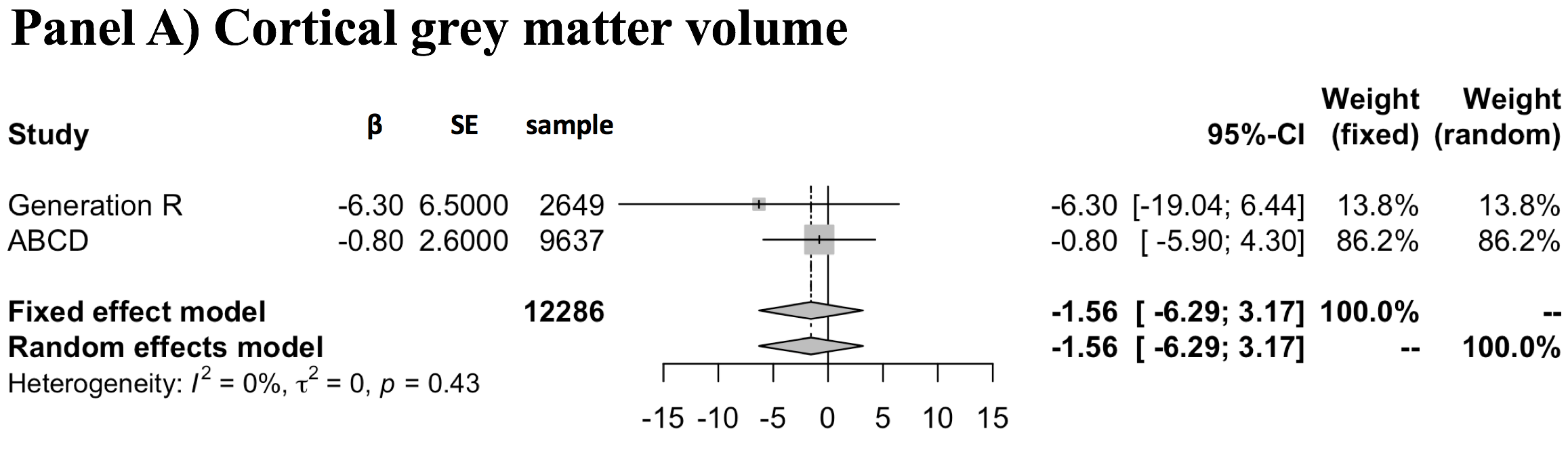

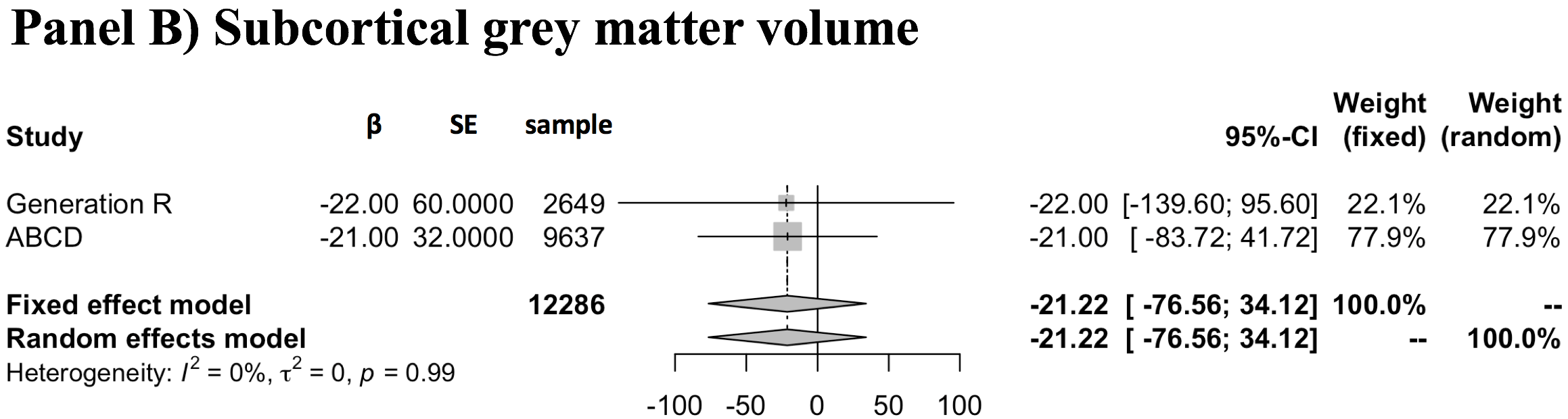

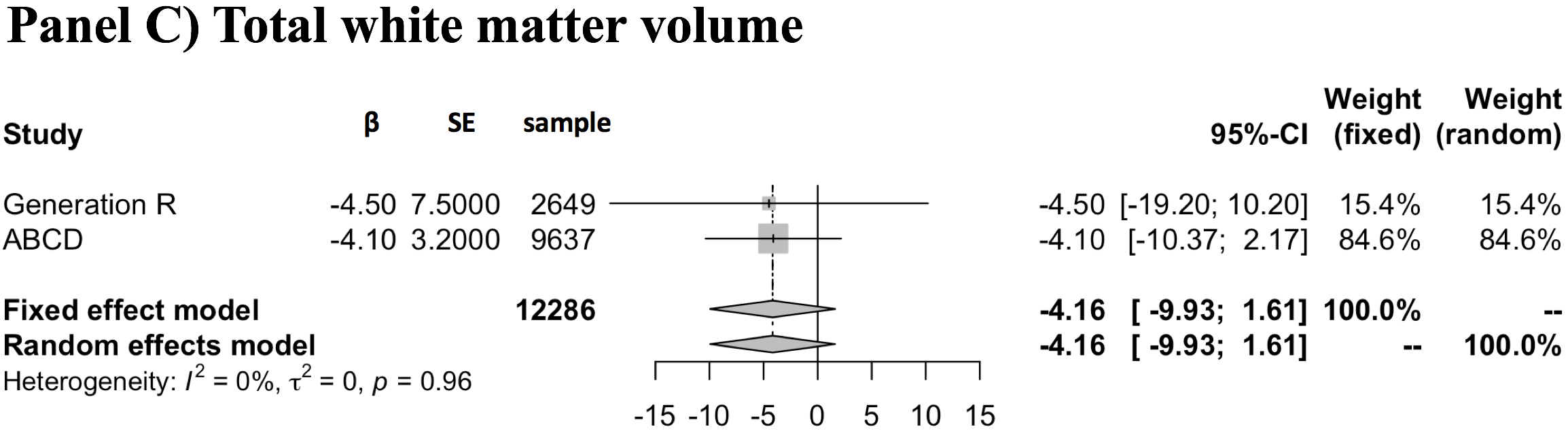


Supplementary figure S1. Meta-analysis of the association between global brain metrics and physical symptoms.

*Note.* β, unstandardised regression coefficient. SE, standard error; CI, Confidence interval; ABCD, Adolescent Brain Cognitive Development.


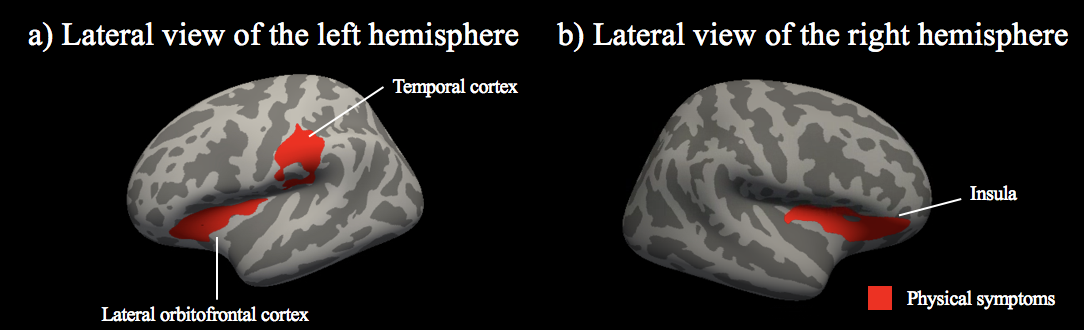


Supplementary figure S2. Significant associations (p_uncorrected_<0.001) between surface area and continuous scores of physical symptoms in the ABCD study (N=9,637).

*Note*. Significant clusters of < 45mm^2^ are not annotated in the figure because they were considered exceedingly small. The models were adjusted for age, sex, race/ethnicity, estimated intracranial volume, parental education, household income, body mass index, non-verbal intelligence quotient and the 21 study sites. Physical symptoms were assessed using the school-age version (for ages 6 to 18) of the Child Behavior Checklist (CBCL). The medial view of the brain is not showed because significant associations did not emerge.


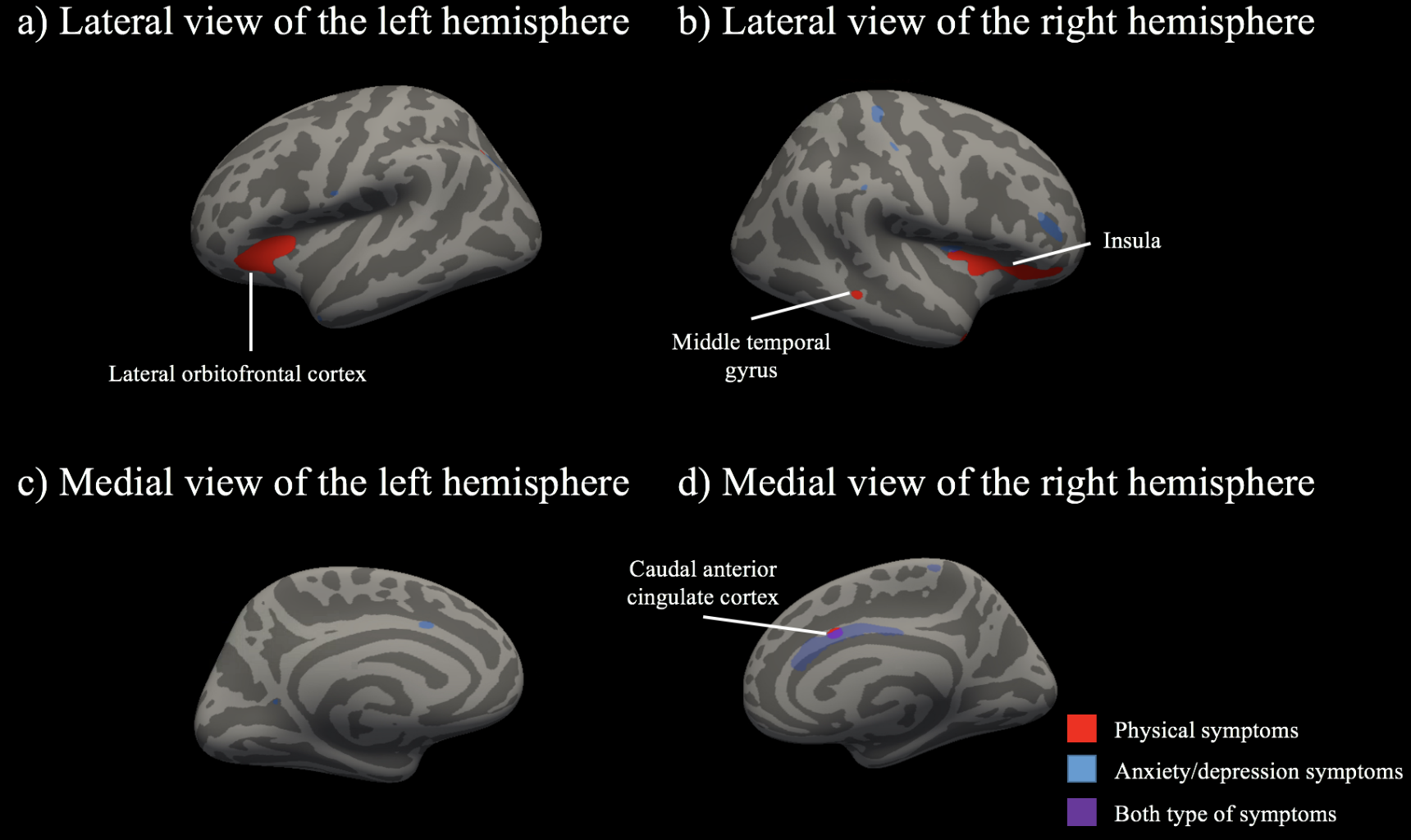


Supplementary figure S3. Significant associations (p_uncorrected_<0.001) between surface area and dichotomous scores of physical symptoms, anxiety/depression symptoms and both types of symptoms from meta-analyses without correcting for multiple testing (N=12,286).

*Note*. Significant clusters of < 45mm^2^ are not annotated in the figure because they were considered exceedingly small. Specific findings for anxiety/depression symptoms are not annotated because are not the primary focus of this work. The models were adjusted for age, sex, national origin (Generation R) or race/ethnicity (ABCD), estimated intracranial volume, maternal education (Generation R) or parental education (ABCD), household income, body mass index and non-verbal intelligence quotient. ABCD analyses were additionally adjusted for the 21 study sites. Physical symptoms were assessed using the school-age version (for ages 6 to 18) of the Child Behavior Checklist (CBCL).
